# 3-(3-Chloro­prop­yl)-7,8-dimeth­oxy-1*H*-3-benzazepin-2(3*H*)-one at 125 K

**DOI:** 10.1107/S1600536808011264

**Published:** 2008-04-26

**Authors:** Xiang-Wei Cheng

**Affiliations:** a Zhejiang Police College Experience Center, Zhejiang Police College, Hangzhou 310053, People’s Republic of China

## Abstract

In the title compound, C_15_H_18_ClNO_3_, the seven-membered ring has a mirror plane passing through the methyl­ene C atom and bis­ecting the C=C bond. It adopts a bent conformation, inter­mediate between the boat and chair forms. Both meth­oxy groups are coplanar with the attached benzene ring [C—C—O—C = −0.5 (3) and 2.2 (3)°]. In the crystal structure, inversion-related mol­ecules are linked *via* C—H⋯O hydrogen bonds and π–π inter­actions involving the benzene ring [centroid–centroid distance = 3.6393 (12)Å].

## Related literature

For the synthesis, see: Reiffen *et al.* (1990 ▶). For general background, see: Franke *et al.* (1987 ▶); Ishihara *et al.* (1994 ▶). For a related structure, see: Cheng (2008 ▶). For asymmetry parameters, see: Duax *et al.* (1976 ▶).
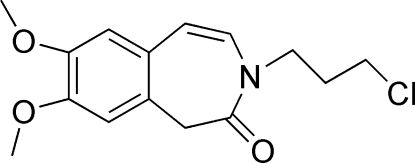


## Experimental

### 

#### Crystal data

C_15_H_18_ClNO_3_
*M* *_r_* = 295.75
Triclinic, 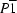

*a* = 9.3141 (17) Å
*b* = 9.5924 (17) Å
*c* = 9.6359 (17) Å
α = 103.667 (6)°
β = 114.701 (6)°
γ = 94.460 (6)°
*V* = 744.7 (2) Å^3^
*Z* = 2
Mo *K*α radiation
μ = 0.26 mm^−1^
*T* = 123 (2) K
0.29 × 0.26 × 0.21 mm

#### Data collection

Bruker SMART CCD area-detector diffractometer
Absorption correction: multi-scan (*SADABS*; Bruker, 2002 ▶) *T*_min_ = 0.928, *T*_max_ = 0.947
7023 measured reflections
2574 independent reflections
2092 reflections with *I* > 2σ(*I*)
*R*_int_ = 0.019

#### Refinement

*R*[*F* ^2^ > 2σ(*F* ^2^)] = 0.043
*wR*(*F* ^2^) = 0.115
*S* = 1.01
2574 reflections
181 parameters
H-atom parameters constrained
Δρ_max_ = 0.21 e Å^−3^
Δρ_min_ = −0.34 e Å^−3^

### 

Data collection: *SMART* (Bruker, 2002 ▶); cell refinement: *SAINT* (Bruker, 2002 ▶); data reduction: *SAINT*; program(s) used to solve structure: *SHELXS97* (Sheldrick, 2008 ▶); program(s) used to refine structure: *SHELXL97* (Sheldrick, 2008 ▶); molecular graphics: *SHELXTL* (Sheldrick, 2008 ▶); software used to prepare material for publication: *SHELXTL*.

## Supplementary Material

Crystal structure: contains datablocks global, I. DOI: 10.1107/S1600536808011264/ci2582sup1.cif

Structure factors: contains datablocks I. DOI: 10.1107/S1600536808011264/ci2582Isup2.hkl

Additional supplementary materials:  crystallographic information; 3D view; checkCIF report

## Figures and Tables

**Table 1:** Hydrogen-bond geometry (Å, °)

*D*—H⋯*A*	*D*—H	H⋯*A*	*D*⋯*A*	*D*—H⋯*A*
C3—H3⋯O3^i^	0.93	2.56	3.473 (2)	169

Symmetry code: (i) 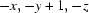.

**Fig. 1.**
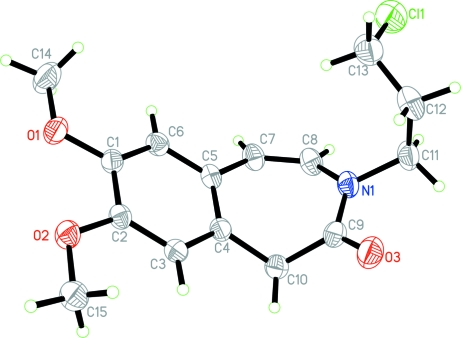
Molecular structure of the title compound, showing 30% probability displacement ellipsoids and the atomic numbering.

**Fig. 2.**
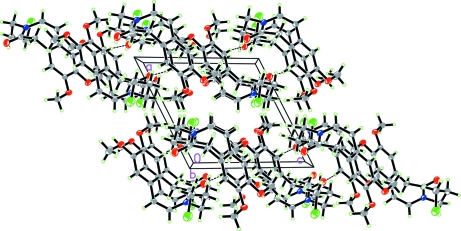
Crystal packing of the title compound, viewed approximately down the b axis. Dashed lines indicate intermolecular hydrogen bonds.

## supplementary crystallographic information

### Comment

Benzazepine derivatives have been of considerable medicinal interest, partly because the skeleton is a component of amaryllydaceae alkaloids such as galanthamine as well as of ribasine alkaloids represented by ribasine (Ishihara et al., 1994). Many benzazepine derivatives have been reported to possess interesting biological activities. The title compound is an important intermediate of ivabradine, which was listed in market in 2006 as the representative of a novel pharmacological class termed reducing heart rate without concomitant negative inotropic or hypotensive effects (Franke et al., 1987). Rencently, in our previous research, a crystal structure of another important intermediate of ivabradine, 7,8-Dimethoxy-3-(3-chloropropyl)- 2,3,4,5-tetrahydro-1H-3-benzazepin-2-one was reported (Cheng, 2008). Here the crystal structure of the title compound is reported.

In the title molecule (Fig.1), the seven-membered ring adopts a bent conformation, intermediate between the boat and chair forms. The seven-membered ring possesses a mirror symmetry about the plane passing through atom C10 and bisecting the C7—C8 bond. The asymmetry parameter (Duax et al., 1976), ΔC_s_(C10), is 2.9 (2)°. The dihedral angle between the C1-C7/C10/C14/C15/O1/O2 and C8-C11/N1/O3 planes is 59.17 (6)°. The chloropropyl substituent group is in a (-)-synclinal conformation, as evidenced by the torsion angle N1—C11—C12—C13 of -63.4 (3)°, similar to that in a related structure (-68.9 (2)°, Cheng, 2008). The methoxy groups are coplanar with the benzene ring [C14—C1—O1—C6 = -0.5 (3)° and C15—C2—O2—C3 = 2.2 (3)°].

An intermolecular C—H···O hydrogen bond is observed in the crystal structure. Also, a π-π interaction involving the benzene ring is observed [centroid to centroid distance = 3.6393 (12) Å].

### Experimental

The title compound was prepared according to the literature method (Reiffen *et al.*, 1990). Crystals suitable for X-ray analysis were obtained by slow evaporation of an ethanol solution at 295 K.

### Refinement

H atoms were positioned geometrically (C-H = 0.93-0.97 Å) and refined using a riding model, with *U*_iso_(H) = 1.2–1.5*U*_eq_(C).

### Crystal data

**Table:**

C_15_H_18_ClNO_3_	*Z* = 2
*M**_r_* = 295.75	*F*_000_ = 312
Triclinic, *P*1	*D*_x_ = 1.319 Mg m^−^^3^
Hall symbol: -P 1	Mo *K*α radiation λ = 0.71073 Å
*a* = 9.3141 (17) Å	Cell parameters from 2574 reflections
*b* = 9.5924 (17) Å	θ = 2.2–25.0º
*c* = 9.6359 (17) Å	µ = 0.26 mm^−^^1^
α = 103.667 (6)º	*T* = 123 (2) K
β = 114.701 (6)º	Block, yellow
γ = 94.460 (6)º	0.29 × 0.26 × 0.21 mm
*V* = 744.7 (2) Å^3^	

### Data collection

**Table:**

Bruker SMART CCD area-detector diffractometer	2574 independent reflections
Radiation source: fine-focus sealed tube	2092 reflections with *I* > 2σ(*I*)
Monochromator: graphite	*R*_int_ = 0.019
*T* = 123(2) K	θ_max_ = 25.0º
φ and ω scans	θ_min_ = 2.2º
Absorption correction: multi-scan(SADABS; Bruker, 2002)	*h* = −11→11
*T*_min_ = 0.928, *T*_max_ = 0.947	*k* = −11→10
7023 measured reflections	*l* = −11→11

### Refinement

**Table:**

Refinement on *F*^2^	Secondary atom site location: difference Fourier map
Least-squares matrix: full	Hydrogen site location: inferred from neighbouring sites
*R*[*F*^2^ > 2σ(*F*^2^)] = 0.043	H-atom parameters constrained
*wR*(*F*^2^) = 0.115	*w* = 1/[σ^2^(*F*_o_^2^) + (0.0542*P*)^2^ + 0.2173*P*] where *P* = (*F*_o_^2^ + 2*F*_c_^2^)/3
*S* = 1.02	(Δ/σ)_max_ = 0.001
2574 reflections	Δρ_max_ = 0.21 e Å^−^^3^
181 parameters	Δρ_min_ = −0.34 e Å^−^^3^
Primary atom site location: structure-invariant direct methods	Extinction correction: none

### Special details

**Table:**

Geometry. All esds (except the esd in the dihedral angle between two l.s. planes) are estimated using the full covariance matrix. The cell esds are taken into account individually in the estimation of esds in distances, angles and torsion angles; correlations between esds in cell parameters are only used when they are defined by crystal symmetry. An approximate (isotropic) treatment of cell esds is used for estimating esds involving l.s. planes.
Refinement. Refinement of F^2^ against ALL reflections. The weighted R-factor wR and goodness of fit S are based on F^2^, conventional R-factors R are based on F, with F set to zero for negative F^2^. The threshold expression of F^2^ > 2sigma(F^2^) is used only for calculating R-factors(gt) etc. and is not relevant to the choice of reflections for refinement. R-factors based on F^2^ are statistically about twice as large as those based on F, and R- factors based on ALL data will be even larger.

### Fractional atomic coordinates and isotropic or equivalent isotropic displacement parameters (Å^2^)

**Table:**

	*x*	*y*	*z*	*U*_iso_*/*U*_eq_	
Cl1	0.40282 (11)	−0.14655 (9)	0.18804 (11)	0.1048 (3)	
O1	−0.20936 (16)	0.23306 (15)	0.46481 (17)	0.0598 (4)	
O2	−0.31187 (14)	0.39739 (15)	0.28346 (16)	0.0535 (3)	
O3	0.15852 (19)	0.32805 (18)	−0.03080 (19)	0.0722 (4)	
N1	0.31547 (18)	0.24927 (17)	0.17583 (19)	0.0513 (4)	
C2	−0.16173 (19)	0.37032 (18)	0.3119 (2)	0.0422 (4)	
C4	0.0877 (2)	0.39343 (19)	0.2909 (2)	0.0445 (4)	
C6	0.0472 (2)	0.2518 (2)	0.4511 (2)	0.0476 (4)	
H6	0.0858	0.1949	0.5196	0.057*	
C1	−0.1044 (2)	0.28198 (19)	0.4125 (2)	0.0447 (4)	
C10	0.1945 (2)	0.4592 (2)	0.2308 (3)	0.0557 (5)	
H10A	0.2980	0.5093	0.3203	0.067*	
H10B	0.1452	0.5306	0.1797	0.067*	
C3	−0.0662 (2)	0.42458 (19)	0.2514 (2)	0.0450 (4)	
H3	−0.1046	0.4823	0.1839	0.054*	
C5	0.1450 (2)	0.30533 (19)	0.3888 (2)	0.0455 (4)	
C7	0.3048 (2)	0.2705 (2)	0.4297 (2)	0.0536 (5)	
H7	0.3613	0.2625	0.5321	0.064*	
C9	0.2193 (2)	0.3412 (2)	0.1127 (3)	0.0540 (5)	
C8	0.3789 (2)	0.2489 (2)	0.3359 (2)	0.0556 (5)	
H8	0.4831	0.2318	0.3815	0.067*	
C11	0.3610 (2)	0.1450 (2)	0.0693 (3)	0.0623 (6)	
H11A	0.4657	0.1257	0.1324	0.075*	
H11B	0.3707	0.1894	−0.0078	0.075*	
C15	−0.3741 (2)	0.4838 (2)	0.1797 (3)	0.0607 (5)	
H15A	−0.4794	0.4958	0.1684	0.091*	
H15B	−0.3814	0.4361	0.0768	0.091*	
H15C	−0.3037	0.5780	0.2234	0.091*	
C14	−0.1591 (3)	0.1435 (3)	0.5654 (4)	0.0892 (8)	
H14A	−0.2426	0.1169	0.5934	0.134*	
H14B	−0.0630	0.1957	0.6607	0.134*	
H14C	−0.1373	0.0566	0.5107	0.134*	
C12	0.2402 (3)	0.0012 (3)	−0.0196 (3)	0.0729 (7)	
H12A	0.1358	0.0215	−0.0819	0.088*	
H12B	0.2723	−0.0581	−0.0940	0.088*	
C13	0.2220 (3)	−0.0865 (3)	0.0846 (3)	0.0822 (7)	
H13A	0.1351	−0.1710	0.0188	0.099*	
H13B	0.1932	−0.0270	0.1618	0.099*	

### Atomic displacement parameters (Å^2^)

**Table:**

	*U*^11^	*U*^22^	*U*^33^	*U*^12^	*U*^13^	*U*^23^
Cl1	0.1226 (6)	0.1022 (6)	0.1229 (7)	0.0475 (5)	0.0735 (5)	0.0491 (5)
O1	0.0562 (8)	0.0706 (9)	0.0752 (10)	0.0200 (7)	0.0406 (7)	0.0384 (8)
O2	0.0394 (7)	0.0670 (8)	0.0629 (8)	0.0175 (6)	0.0249 (6)	0.0286 (7)
O3	0.0810 (10)	0.0901 (11)	0.0677 (10)	0.0291 (9)	0.0454 (9)	0.0361 (9)
N1	0.0452 (8)	0.0567 (9)	0.0566 (10)	0.0119 (7)	0.0297 (8)	0.0114 (7)
C2	0.0367 (9)	0.0438 (9)	0.0426 (10)	0.0078 (7)	0.0173 (8)	0.0078 (8)
C4	0.0442 (9)	0.0422 (9)	0.0490 (10)	0.0096 (7)	0.0248 (8)	0.0092 (8)
C6	0.0506 (10)	0.0504 (10)	0.0446 (10)	0.0176 (8)	0.0214 (8)	0.0164 (8)
C1	0.0455 (9)	0.0452 (10)	0.0460 (10)	0.0091 (8)	0.0240 (8)	0.0112 (8)
C10	0.0543 (11)	0.0504 (11)	0.0753 (14)	0.0134 (9)	0.0394 (10)	0.0208 (10)
C3	0.0485 (10)	0.0426 (9)	0.0468 (10)	0.0129 (8)	0.0232 (8)	0.0141 (8)
C5	0.0428 (9)	0.0477 (10)	0.0449 (10)	0.0115 (8)	0.0221 (8)	0.0067 (8)
C7	0.0459 (10)	0.0654 (12)	0.0476 (11)	0.0191 (9)	0.0196 (9)	0.0135 (9)
C9	0.0480 (10)	0.0581 (12)	0.0679 (14)	0.0088 (9)	0.0358 (10)	0.0216 (10)
C8	0.0384 (9)	0.0649 (12)	0.0582 (12)	0.0154 (9)	0.0199 (9)	0.0111 (9)
C11	0.0614 (12)	0.0692 (13)	0.0696 (14)	0.0187 (10)	0.0442 (11)	0.0145 (11)
C15	0.0469 (11)	0.0752 (14)	0.0683 (13)	0.0253 (10)	0.0250 (10)	0.0335 (11)
C14	0.0905 (18)	0.111 (2)	0.121 (2)	0.0422 (15)	0.0707 (17)	0.0788 (19)
C12	0.0688 (14)	0.0743 (15)	0.0642 (14)	0.0169 (11)	0.0305 (12)	−0.0015 (12)
C13	0.0774 (16)	0.0664 (14)	0.0969 (19)	−0.0032 (12)	0.0500 (15)	−0.0006 (13)

### Geometric parameters (Å, °)

**Table:**

Cl1—C13	1.783 (3)	C3—H3	0.93
O1—C1	1.372 (2)	C5—C7	1.460 (2)
O1—C14	1.405 (3)	C7—C8	1.338 (3)
O2—C2	1.367 (2)	C7—H7	0.93
O2—C15	1.416 (2)	C8—H8	0.93
O3—C9	1.225 (2)	C11—C12	1.515 (3)
N1—C9	1.368 (3)	C11—H11A	0.97
N1—C8	1.404 (3)	C11—H11B	0.97
N1—C11	1.476 (2)	C15—H15A	0.96
C2—C3	1.381 (2)	C15—H15B	0.96
C2—C1	1.403 (2)	C15—H15C	0.96
C4—C5	1.384 (3)	C14—H14A	0.96
C4—C3	1.398 (2)	C14—H14B	0.96
C4—C10	1.510 (3)	C14—H14C	0.96
C6—C1	1.375 (2)	C12—C13	1.504 (4)
C6—C5	1.409 (3)	C12—H12A	0.97
C6—H6	0.93	C12—H12B	0.97
C10—C9	1.510 (3)	C13—H13A	0.97
C10—H10A	0.97	C13—H13B	0.97
C10—H10B	0.97		
			
C1—O1—C14	117.58 (15)	N1—C9—C10	115.85 (18)
C2—O2—C15	116.59 (14)	C7—C8—N1	126.72 (17)
C9—N1—C8	125.16 (16)	C7—C8—H8	116.6
C9—N1—C11	117.85 (17)	N1—C8—H8	116.6
C8—N1—C11	116.96 (16)	N1—C11—C12	112.93 (16)
O2—C2—C3	124.88 (16)	N1—C11—H11A	109.0
O2—C2—C1	115.42 (15)	C12—C11—H11A	109.0
C3—C2—C1	119.69 (15)	N1—C11—H11B	109.0
C5—C4—C3	120.30 (16)	C12—C11—H11B	109.0
C5—C4—C10	119.48 (15)	H11A—C11—H11B	107.8
C3—C4—C10	120.20 (16)	O2—C15—H15A	109.5
C1—C6—C5	121.25 (17)	O2—C15—H15B	109.5
C1—C6—H6	119.4	H15A—C15—H15B	109.5
C5—C6—H6	119.4	O2—C15—H15C	109.5
O1—C1—C6	125.56 (16)	H15A—C15—H15C	109.5
O1—C1—C2	114.89 (15)	H15B—C15—H15C	109.5
C6—C1—C2	119.54 (16)	O1—C14—H14A	109.5
C4—C10—C9	110.38 (15)	O1—C14—H14B	109.5
C4—C10—H10A	109.6	H14A—C14—H14B	109.5
C9—C10—H10A	109.6	O1—C14—H14C	109.5
C4—C10—H10B	109.6	H14A—C14—H14C	109.5
C9—C10—H10B	109.6	H14B—C14—H14C	109.5
H10A—C10—H10B	108.1	C13—C12—C11	115.1 (2)
C2—C3—C4	120.54 (16)	C13—C12—H12A	108.5
C2—C3—H3	119.7	C11—C12—H12A	108.5
C4—C3—H3	119.7	C13—C12—H12B	108.5
C4—C5—C6	118.65 (15)	C11—C12—H12B	108.5
C4—C5—C7	120.94 (17)	H12A—C12—H12B	107.5
C6—C5—C7	120.40 (17)	C12—C13—Cl1	111.93 (17)
C8—C7—C5	127.12 (18)	C12—C13—H13A	109.2
C8—C7—H7	116.4	Cl1—C13—H13A	109.2
C5—C7—H7	116.4	C12—C13—H13B	109.2
O3—C9—N1	121.47 (18)	Cl1—C13—H13B	109.2
O3—C9—C10	122.68 (19)	H13A—C13—H13B	107.9
			
C15—O2—C2—C3	2.2 (3)	C10—C4—C5—C7	−2.4 (3)
C15—O2—C2—C1	−178.74 (16)	C1—C6—C5—C4	1.9 (3)
C14—O1—C1—C6	−0.5 (3)	C1—C6—C5—C7	−179.24 (17)
C14—O1—C1—C2	179.7 (2)	C4—C5—C7—C8	−35.3 (3)
C5—C6—C1—O1	178.90 (16)	C6—C5—C7—C8	145.9 (2)
C5—C6—C1—C2	−1.3 (3)	C8—N1—C9—O3	−174.47 (18)
O2—C2—C1—O1	1.4 (2)	C11—N1—C9—O3	7.6 (3)
C3—C2—C1—O1	−179.52 (15)	C8—N1—C9—C10	5.9 (3)
O2—C2—C1—C6	−178.43 (15)	C11—N1—C9—C10	−172.03 (16)
C3—C2—C1—C6	0.7 (3)	C4—C10—C9—O3	109.0 (2)
C5—C4—C10—C9	68.9 (2)	C4—C10—C9—N1	−71.4 (2)
C3—C4—C10—C9	−112.88 (19)	C5—C7—C8—N1	−2.8 (4)
O2—C2—C3—C4	178.40 (16)	C9—N1—C8—C7	37.8 (3)
C1—C2—C3—C4	−0.6 (3)	C11—N1—C8—C7	−144.2 (2)
C5—C4—C3—C2	1.2 (3)	C9—N1—C11—C12	−88.5 (2)
C10—C4—C3—C2	−176.99 (16)	C8—N1—C11—C12	93.4 (2)
C3—C4—C5—C6	−1.8 (3)	N1—C11—C12—C13	−63.4 (3)
C10—C4—C5—C6	176.41 (16)	C11—C12—C13—Cl1	−64.6 (2)
C3—C4—C5—C7	179.33 (16)		

### Hydrogen-bond geometry (Å, °)

**Table:**

*D*—H···*A*	*D*—H	H···*A*	*D*···*A*	*D*—H···*A*
C3—H3···O3^i^	0.93	2.56	3.473 (2)	169

Symmetry codes: (i) −*x*, −*y*+1, −*z*.
